# Global Magnitude of Reported and Unreported Mesothelioma

**DOI:** 10.1289/ehp.1002845

**Published:** 2011-01-06

**Authors:** Eun-Kee Park, Ken Takahashi, Tsutomu Hoshuyama, Tsun-Jen Cheng, Vanya Delgermaa, Giang Vinh Le, Tom Sorahan

**Affiliations:** 1 Department of Environmental Epidemiology, Institute of Industrial Ecological Sciences, University of Occupational and Environmental Health, Kitakyushu City, Japan; 2 Institute of Occupational Medicine and Industrial Hygiene, College of Public Health, National Taiwan University, Taipei, Taiwan; 3 Institute of Occupational and Environmental Medicine, University of Birmingham, Edgbaston, Birmingham, United Kingdom

**Keywords:** asbestos, frequency, mesothelioma, mortality, prediction

## Abstract

**Background:**

Little is known about the global magnitude of mesothelioma. In particular, many developing countries, including some with extensive historical use of asbestos, do not report mesothelioma.

**Objectives:**

We estimated the global magnitude of mesothelioma accounting for reported and unreported cases.

**Methods:**

For all countries with available data on mesothelioma frequency and asbestos use (*n* = 56), we calculated the 15-year cumulative number of mesotheliomas during 1994–2008 from data available for fewer years and assessed its relationship with levels of cumulative asbestos use during 1920–1970. We used this relationship to predict the number of unreported mesotheliomas in countries for which no information on mesothelioma is available but which have recorded asbestos use (*n* = 33).

**Results:**

Within the group of 56 countries with data on mesothelioma occurrence and asbestos use, the 15-year cumulative number of mesothelioma was approximately 174,300. There was a statistically significant positive linear relation between the log-transformed national cumulative mesothelioma numbers and the log-transformed cumulative asbestos use (adjusted *R*^2^ = 0.83, *p* < 0.0001). Extrapolated to the group of 33 countries without reported mesothelioma, a total of approximately 38,900 (95% confidence interval, 36,700–41,100) mesothelioma cases were estimated to have occurred in the 15-year period (1994–2008).

**Conclusions:**

We estimate conservatively that, globally, one mesothelioma case has been overlooked for every four to five reported cases. Because our estimation is based on asbestos use until 1970, the many countries that increased asbestos use since then should anticipate a higher disease burden in the immediate decades ahead.

Malignant mesothelioma is a major public health concern, because this rare form of cancer—caused specifically by exposure to asbestos—is difficult to diagnose, has extremely poor prognosis, and is on the increase. Epidemics of mesothelioma have been reported nationally (Centers for Disease Control and Prevention 2009; Hodgson et al. 2005; Kjellstrom and Smartt 2000; Murayama et al. 2006) and regionally (Pelucchi et al. 2004; Peto et al. 1999), but available information is biased toward developed countries and regions (referred to hereafter as “countries”) with the resources to diagnose asbestos-related diseases (ARDs) and with known historical use of asbestos. At present, mesothelioma is grossly underreported in many developing countries (LaDou 2004; Takahashi and Karjalainen 2003), including some with known extensive use of asbestos.

Few attempts have been made to quantify the global incidence of mesothelioma. In a study of the total burden of occupational carcinogens, Driscoll et al. (2005) reported 43,000 mesothelioma deaths/year, based on an estimated proportion of exposed workers and levels of exposure, combined with absolute risk measures. This number, endorsed in a World Health Organization (WHO) document on the elimination of ARDs (WHO 2006a), is widely quoted to guide preventive activities. However, there has been no validation or reassessment of this 2005 estimate, possibly because the indices that were used are difficult to access and reproduce. Commonly available statistics should be used to address the shortage of information, which may also improve estimates of the disease burden.

In a previous study (Lin et al. 2007), we calculated the volume of asbestos consumed per head (per-capita asbestos use) from a report by the U.S. Geological Survey (USGS) (Virta 2006) and used this value as a surrogate for population exposure level. Our results indicated that rates of past asbestos use can predict recent death rates from four types of ARDs at national levels and explained the bulk of the variance (Lin et al. 2007). This relationship, albeit ecological, is likely to reflect a causal relationship. Other researchers have used this surrogate indicator to estimate or predict ARDs in different populations (Antao et al. 2009; Tse et al. 2010).

Information related to mesothelioma frequency is accumulating in relation to the growing number of national and regional registries (Leigh and Driscoll 2003; Marinaccio et al. 2007). In the present study, we estimated the magnitude of mesothelioma in the world accounting for both reported and unreported numbers by using a global database and extending our previous ecological model. We incorporated updated data from the widest possible sources of information, assuming that mortality reflects incidence for this fatal form of cancer. We employed cumulative indicators of asbestos use and number of mesothelioma cases, hypothesizing that recent national burden of mesothelioma is a consequence of historical cumulative use of asbestos. This relationship was then applied to countries that lack relevant health data.

## Materials and Methods

We extracted all data on asbestos use from a report by the USGS (Virta 2006, 2009). We adopted the USGS definition of use (production plus import minus export), the data for which are available by country, in 10-year intervals from 1920 to 1970, in 5-year intervals from 1970 to 1995, and annually from 1995 to 2007. We treated a reported negative value of asbestos use (caused by storage, for example) as zero in this analysis. Using linear interpolation, cumulative asbestos use was calculated independently for two periods (1920–1970 and 1971–2007) to allow a sufficient lag time from the earlier period to that of mesothelioma observation (1994–2008). When necessary, we interpolated values for asbestos use for the calendar year lacking data.

The cumulative numbers of all types of mesothelioma [code C45; *International Classification of Diseases, 10th Revision* (ICD-10; WHO 2006a b] were obtained from the WHO Mortality Database (WHO 2010a) and tallied by country. Note that the WHO data comprise deaths registered in national civil registration systems, with underlying cause of death as coded by the relevant national authority (WHO 2010b). Data were extracted for countries with at least 3 years of data coded as C45 (ICD-10) or any of its subcategories. To maximize use of available data, we separately counted numbers recorded for malignant neoplasm of the pleura (code 163; *International Classification of Diseases, 9th Revision* (ICD-9; WHO 1977)]. To investigate countries that did not report data to the WHO, we used PubMed (National Center for Biotechnology Information 2010) and other sources to search for national frequency data published in English (Lee et al. 2010; Rüegger 2008). Data were prioritized for analysis in that order, and overlapping information was evaluated once only. For a number of countries, national counts of mesothelioma deaths were reported for intermittent years or did not span the entire period. For each country, the 15-year cumulative number was estimated by first calculating the annual mean of reported mesothelioma deaths from data available for fewer years, which was then multiplied by 15.

National population data were obtained from the WHO (2010a) and the U.S. Census Bureau (2010), and prioritized for use in that order.

Of the 89 analyzed countries, 12 warranted special treatment of asbestos use data because of political transition (e.g., disintegration, unification) or combined treatment with other countries/entities by the USGS. We used historical information in an unbiased manner to the extent possible to give continuity with data of countries existing today:

- Data on asbestos use for the Soviet Union during 1920–1990 in the USGS database represented those of Russia and Kazakhstan combined (Virta 2006). We thus apportioned data recorded by the Soviet Union during 1920–1990 between Russia and Kazakhstan during 1920–1990 according to the ratio of use recorded by Russia and Kazakhstan during 1995–2007.
- Data on asbestos use for West and East Germany during 1950–1985 were combined into one entity (i.e., Germany).
- To account for the disintegration of Czechoslovakia in 1993 (United Nations 2010), data on asbestos use for Czechoslovakia during 1920–1990 were apportioned to the level of use during 1995–2007 between Czech Republic and Slovakia.
- To account for the disintegration of Yugoslavia in 1991 (Duffield 2003), data on asbestos use for Yugoslavia during 1930–1990 were apportioned to the level of use during 1995–2007 among Bosnia and Herzegovina, Croatia, Serbia and Montenegro, the former Yugoslav Republic of Macedonia, and Slovenia (we treated Serbia and Montenegro as one entity).
- Data on asbestos use for Belgium and Luxembourg were combined during 1930–2001 in the USGS database (Virta 2006). We apportioned these data to Belgium and Luxembourg according to the size of the respective populations during this period.

We compiled all data and performed descriptive statistics using Microsoft Excel (Microsoft Corporation, Redmond, WA, USA). We then conducted the regression analyses using SAS, version 9.1 (SAS Institute Inc., Cary, NC, USA). Because of the extremely wide dispersion of data for both asbestos and mesothelioma, data were log-transformed to construct the regression model: log_10_ (mortality) = *B*_0_ + [*B*_1_ × log_10_ (asbestos)], where *B*_0_ is the intercept and *B*_1_ is the coefficient. We applied PROC REG (SAS Institute Inc.) to obtain the parameters and their confidence limits, the adjusted *R*^2^, and *p*-values of the regression model. The size of the national populations was used as weights. To further obtain the confidence limits for the mean predicted value for each observation, we added the confidence limits for the mean option. This accounted for the variation due to estimating the parameters only. All the aforementioned processes were accomplished in one run of PROC REG.

After predicted values were obtained, based on the principle that if *Y_i_* ~ *N*(*m_i_, s_i_*^2^) is the measurement for the *i*th cluster then *Y* = sum(*Y_i_*) ~ *N*[sum(*m_i_*), sum(*s_i_*^2^)], the confidence limits for the sum of predicted values were calculated from the square root of the sum of the variance.

We used Sigmaplot (version 9.01; Systat Software Inc., San Jose, CA, USA) to draw the figure; the size of the bubbles is proportionate to the size of national populations of the year 2000. *p*-Values < 0.05 were deemed statistically significant in all analyses.

## Results

Globally, 89 countries had available information on frequency of mesothelioma and/or use of asbestos at the national level. These countries represented 82.6% of the global population in the year 2000. Of these countries, 56 had data for both mesothelioma frequency and asbestos use, and 33 had no mesothelioma frequency data but had data for asbestos use (Table 1).

The cumulative asbestos use during 1920–1970 was 51.2 million metric tons in the 56 countries having data on both mesothelioma frequency and asbestos use and 14.2 million metric tons in the 33 countries having data on asbestos use only, totaling 65.4 million metric tons in all 89 analyzed countries. This volume represented 100% of the global asbestos use during 1920–1970. By individual country, cumulative asbestos use was highly skewed, led by the United States, Russia, United Kingdom, Germany, and Japan, with volumes at 21.8, 8.4, 4.8, 4.1, and 3.2 million metric tons, respectively (Tables 2 and 3). The 56 countries with mesothelioma data reported a total of 92,133 deaths during 1994–2008 (336 of which were coded as ICD-9 163 by four countries) (Table 1).

Table 2 shows the 15-year cumulative mortality of mesothelioma extrapolated from numbers reported for fewer years in the 56 countries with mesothelioma data, ranked in order of the cumulative asbestos use. The 15-year cumulative numbers are highly skewed but generally paralleled the level of cumulative asbestos use. Leading countries for the 15-year mesothelioma number are the United States (36,561 cases), the United Kingdom (28,369 cases), Italy (18,530 cases), Germany (15,948 cases), and France (12,390 cases) (Table 2). The total 15-year cumulative mortality was approximately 174,300 deaths.

**Figure 1:**
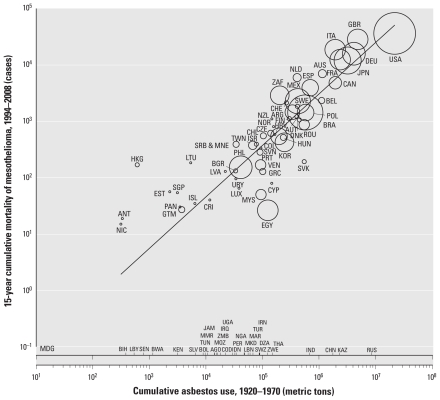
Relationship between 15-year cumulative mortality of mesothelioma (1994–2008) and cumulative use of asbestos (1920–1970) weighted by the size of national populations in 56 countries/entities with data for both mesothelioma and asbestos use. Asbestos use for 33 countries/entities without mesothelioma frequency data is indicated along the *x*-axis. The figure is based on the following regression model: log_10_(15-year cumulative mortality of mesothelioma) = β_0_ + β_1_ × log_10_(cumulative use of asbestos), where β_0_ = −1.998 (95% CI, −2.676 to −1.319) and β_1_ = 0.913 (95% CI, 0.800 to 1.026). Adjusted *R*^2^ = 0.827; *p* < 0.0001.

Figure 1 shows scatterplots of national data for the group of 56 countries. There is a clear positive linear relation between the log-transformed values of the 15-year cumulative mortality of mesothelioma during 1994–2008 and the cumulative use of asbestos during 1920–1970. Cumulative asbestos use was a significant predictor of cumulative mesothelioma mortality, with an adjusted *R*^2^ value of 0.83 (*p* < 0.0001). Similar findings were obtained when data for the four countries reporting deaths coded to the ICD-9 were omitted, with an adjusted *R*^2^ value of 0.82 (*p* < 0.0001).

Table 3 shows the predicted 15-year cumulative mortality of mesothelioma in the group of 33 countries that lacked data on mesothelioma frequency; predictions were obtained from the value of cumulative asbestos use applied to the relationship obtained earlier. The range of cumulative asbestos use of the 33 countries, except for Madagascar, fell within the range reported by the 56 countries. Leading countries for the predicted 15-year mesothelioma number are Russia (21,300 cases), Kazakhstan (6,500 cases), China (5,100 cases), India (2,200 cases), and Thailand (500 cases). In total, an estimated 38,900 [95% confidence interval (CI), 36,700–41,100] mesothelioma cases possibly occurred but were unreported during 1994–2008.

## Discussion

It is unlikely that mesothelioma is absent in countries that have used asbestos but do not report mesothelioma frequency. There is increasing evidence that the extent of asbestos use can be used to predict subsequent incidence and mortality of ARDs at national levels (Antao et al. 2009; Lin et al. 2007; Nishikawa et al. 2008; Tse et al. 2010). This is not unexpected, as mesothelioma is almost exclusively attributable to past asbestos exposure. We thus postulated that national experiences would follow reasonably similar patterns where countries lacking mesothelioma data were probably “missing” the disease burden to an extent proportionate to the level of historical cumulative asbestos use. Based on available data of 56 countries, we observed that recent cumulative mortality of mesothelioma is closely related to historical cumulative use of asbestos. Further extrapolation of this relation to the 33 countries with no available data for mesothelioma suggested that one mesothelioma case is unreported for every four to five cases reported worldwide (38,900 unreported vs. 174,300 reported).

Accounting for the reported and unreported numbers, we estimated the global burden of mesothelioma to be 213,200 (15-year cumulative mortality during 1994–2008). This is equivalent to an annual average of approximately 14,200 cases, assuming a flat change rate, or approximately 25,000 cases in the year 2008 assuming a 10% annual increase rate (38,000 cases assuming a 20% annual increase rate). These estimates are larger than the estimated 10,000 mesothelioma deaths proposed by consensus for only the developed regions in the world (Tossavainen 1997) but smaller than the 43,000 mesothelioma deaths estimated for the world by Driscoll et al. (2005). Nevertheless, our values are reasonably close to those of earlier reports, despite the different methods used.

From the observed linear relation between log-transformed values of cumulative mesothelioma and asbestos, *y* = 10^(0.913 * log^ *^x^* ^− 1.998)^, where *y* (cases) is the 15-year cumulative mesothelioma mortality for the period 1994–2008, and *x* (metric tons) is the cumulative asbestos use during 1920–1970. When values are back-transformed to their original units, there is a linear relation between variables *x* and *y*, each dispersed for a very wide range (i.e., to the power of 10). Further, the amount of asbestos use corresponding to one mesothelioma case (asbestos-to-mesothelioma ratio), or *x/y*, varies depending on the level of *x*. For example, the asbestos-to-mesothelioma ratio is 182–222 metric tons per case for cumulative use of 1,000–10,000 metric tons, and 271–331 metric tons per case for cumulative use of 100,000–1,000,000 metric tons [see Supplemental Material, Table 1 (doi:10.1289/ehp.1002845)]. The values recorded here for the asbestos-to-mesothelioma ratio should be distinguished from a ratio reported by Tossavainen (2004) and referenced widely (fixed value of 170 metric tons per case); that value was derived using data from 11 developed countries with looser definitions and time frame.

The present study is the first to provide a global estimate of missed mesothelioma cases accounting for the experience of countries with data. We directly compared national asbestos use and mesothelioma frequency using cumulative indicators (the denominator population would be common to all calculations of rates); this method had the advantage of directly estimating the disease burden and maximizing use of sparse data. Asbestos has an extremely long industrial life span, and populations are repeatedly exposed during production, maintenance, and abatement. Smoking is unrelated to mesothelioma and thus does not have to be accounted for as a confounder. These facts supported the assumption that cumulative asbestos use causes health effects that are reflected in the cumulative mesothelioma count in populations.

There are, however, obvious limitations in the methods in the present study, as well as potential sources of errors in the information applied. First and foremost, our findings are based on an ecological relation, and thus we do not know the extent to which the consumed amounts of asbestos reflected actual exposure levels of populations. Second, extrapolating from the collective experience of one group of countries to another may introduce bias, especially because the former group consisted of developed and developing countries, whereas the latter group consisted of predominantly developing countries. Third, we did not have information for consumed fiber types (e.g., amphiboles, chrysotile).

Whether and the extent to which the aforementioned limitations and possible errors collectively caused overestimation or underestimation merits further consideration. One potential source of overestimation (pertaining to the extrapolation of data from one group to another) is that developed countries may incur more mesothelioma cases because of an older age composition. However, this factor is probably offset by several potential sources of disease underestimation: *a*) a lack of data on the trade of asbestos-containing products could impose additional risk; *b*) our estimates cannot account for the national experiences of the majority of countries in the world with no information on asbestos use (USGS information on asbestos use may be less complete for the less-developed countries, especially for the earlier years); and *c*) underdiagnosis and underreporting of mesothelioma is also a major problem in developed countries. Therefore, our values should be viewed as conservative estimates.

It is plausible that the hidden burden is substantial in countries with high cumulative use of asbestos, including asbestos-producing countries such as Russia, Kazakhstan, China, and India. Underdiagnosis and/or underreporting may have occurred in these countries because of lack of awareness, knowledge, and resources. It is also possible that, even if mesothelioma cases are diagnosed domestically, frequency numbers are not actively disclosed to the international community because of the increasing number of countries adopting bans on asbestos use (Kazan-Allen 2005) on grounds of public health. Some countries with vested interest in maintaining the production and trade of asbestos may be poorly motivated to acknowledge ARDs. For example, Russia is known for not recognizing asbestosis in its territory (Walgate 2010).

The world nearly doubled cumulative use of asbestos from 65 million metric tons up until 1970, to 124 million metric tons since then. In particular, the group of 33 countries not reporting mesothelioma frequency quintupled asbestos use (Table 1). Individually, the number of countries exceeding the cumulative use of 3.0 million metric tons have increased from five in 1971 (United States, Russia, United Kingdom, Germany, and Japan) to eight: Russia (36.1 million metric tons), China (11.2), Kazakhstan (9.6), Japan (8.1), the United States (6.9), Brazil (5.3), Germany (5.2), India (4.5), and Thailand (3.6) (data not shown). Thus, even with the expected hygienic improvements to reduce exposure over time, and particularly since 1970 onward, these countries should anticipate the need to deal with a very high burden of mesothelioma in the immediate decades ahead.

Developed countries should share experience and technology to enable developing countries to promote accurate diagnosis, reporting, and management of ARDs (Takahashi 2008), including mesothelioma. Moreover, political will is essential to ensure that asbestos use ceases globally.

## Conclusions

We estimated the 15-year cumulative frequency of mesothelioma during 1994–2008 in the 56 countries reporting mesothelioma to be 174,300. Using cumulative asbestos use to predict cumulative mesothelioma frequency at national levels, we predicted the 15-year cumulative frequency of mesothelioma during 1994–2008 in the 33 countries that do not report mesothelioma to be 38,900 (95% CI, 36,700–41,100). Thus, globally, for every four to five reported cases of mesothelioma, one case has been overlooked. These estimates support the need for countermeasures at national, regional, and international levels.

## Figures and Tables

**Table 1:** Baseline characteristics of the 89 analyzed countries/entities with available data for asbestos and/or mesothelioma.

Data category	Both asbestos and mesothelioma (*n =* 56)	Asbestos only (*n =* 33)	Total (*n =* 89)
Asbestos, recorded cumulative use (million metric tons)			
During 1920–1970	51.2	14.2	65.4
During 1971–2007	52.8	71.3	124.1
Mesothelioma, reported cumulative mortality during 1994–2008 [No. of cases (no. of countries)]			
ICD-10, code C45	90,929 (50)	0^a^ (NA)	NA
ICD-9, code 163	336 (4)	0^a^ (NA)	NA
Other data source^b^	868 (2)	0^c^ (NA)	NA
Total	92,133 (56)	0 (33)	NA

NA, not applicable.

a No record in WHO mortality database.

b Published articles in English identified via Pubmed or other source of national data (see References; Lee et al. 2010; Rüegger 2008).

c Data cannot be identified.

**Table 2:** Reported and extrapolated 15-year cumulative mortality of mesothelioma during 1994–2008 in 56 countries/entities with data for mesothelioma mortality and use of asbestos.

	Country (abbreviation)	Cumulative use of asbestos, (tons), 1920–1970	Years with available data (*n*)	Reported cumulative mortality (cases)	Annual average of reported mortality (cases)	Extrapolated 15-year cumulative mortality (cases)
1	USA (USA)	21,840,583	7	17,062	2,437	36,561
2	UK (GBR)	4,829,517	7	13,239	1,891	28,369
3	Germany (DEU)	4,144,825	9	9,569	1,063	15,948
4	Japan (JPN)	3,210,349	14	11,212	801	12,013
5	France (FRA)	2,352,646	8	6,608	826	12,390
6	Canada (CAN)	1,955,347	5	1,603	321	4,809
7	Italy (ITA)	1,934,558	3	3,706	1,235	18,530
8	Australia (AUS)	1,152,776	8	3,747	468	7,026
9	Belgium (BEL)	1,110,214	3	467	156	2,335
10	Spain (ESP)	701,565	7	1,840	263	3,943
11	Poland (POL)	581,013	10	957	96	1,436
12	Brazil (BRA)	577,333	10	955	96	1,433
13	Romania (ROU)	550,799	10	581	58	872
14	Slovakia (SVK)	548,874	12	154	13	193
15	Denmark (DNK)	447,590	13	918	71	1,059
16	Mexico (MEX)	422,645	10	1,513	151	2,270
17	Sweden (SWE)	414,601	11	1,348	123	1,838
18	Netherlands (NLD)	411,989	13	5,141	395	5,932
19	Austria (AUT)	410,249	7	563	80	1,206
20	Argentina (ARG)	338,870	11	1,065	97	1,452
21	Finland (FIN)	299,695	13	970	75	1,119
22	Switzerland (CHE)	267,302	4	568	142	2,130
23	Republic of Korea (KOR)	244,802	12	339	28	424
24	Hungary (HUN)	235,442	13	451	35	520
25	South Africa (ZAF)	203,566	12	2,322	194	2,903
26	Colombia (COL)	196,345	9	323	36	538
27	Croatia (HRV)	165,011	14	547	39	586
28	Norway (NOR)	158,017	12	648	54	810
29	New Zealand (NZL)	147,197	7	513	73	1,099
30	Cyprus (CYP)	145,745	4	21	5	79
31	Czech Republic (CZE)	140,920	15	611	41	611
32	Egypt (EGY)	124,908	4	7	2	26
33	Chile (CHL)	103,780	9	331	37	552
34	Greece (GRC)^a^	101,021	15	128	9	128
35	Malaysia (MYS)	94,540	6	20	3	50
36	Slovenia (SVN)	94,114	12	270	23	338
37	Venezuela (VEN)	93,210	11	124	11	169
38	Portugal (PRT)^a^	90,605	8	152	19	285
39	Israel (ISR)	78,122	10	262	26	393
40	Taiwan (TWN)	67,670	12	300	25	375
41	Philippines (PHL)	41,132	5	51	10	153
42	Luxembourg (LUX)	38,749	9	39	4	65
43	Serbia and Montenegro (SRB & MNE)	34,222	12	313	26	391
44	Uruguay (URY)	33,914	6	38	6	95
45	Bulgaria (BGR)	33,576	4	35	9	131
46	Latvia (LVA)	22,189	13	112	9	129
47	Costa Rica (CRI)	11,718	9	24	3	40
48	Iceland (ISL)	6,417	12	28	2	35
49	Lithuania (LTU)	5,396	11	135	12	184
50	Guatemala (GTM)^a^	3,757	5	9	2	27
51	Panama (PAN)	3,506	7	14	2	30
52	Singapore (SGP)^a^	3,150	13	47	4	54
53	Estonia (EST)	2,300	12	45	4	56
54	Hong Kong (HKG)	616	7	79	11	169
55	Netherlands Antilles (ANT)	335	4	5	1	19
56	Nicaragua (NIC)	316	4	4	1	15
	Total	51,229,638	NA	92,133	NA	174,300

NA, not applicable.

a Number of cases represents malignant neoplasm of the pleura (ICD-9 code 163).

**Table 3:** Predicted 15-year cumulative mortality of mesothelioma in 33 countries/entities with data only for use of asbestos.

	Country (abbreviation)	Cumulative use of asbestos (tons), 1920–1970	Predicted 15-year cumulative mortality (cases)	95% CI
1	Russia (RUS)	8,443,923	21,308	15,026–30,218
2	Kazakhstan (KAZ)	2,301,286	6,500	5,006–8,440
3	China (CHN)	1,767,086	5,107	3,976–6,558
4	India (IND)	688,015	2,158	1,700–2,739
5	Thailand (THA)	152,378	545	400–741
6	Zimbabwe (ZWE)	122,595	447	323–617
7	Algeria (DZA)	90,005	337	238–477
8	Swaziland (SWZ)	87,868	329	232–468
9	Iran (IRN)	68,437	262	181–380
10	Turkey (TUR)	60,345	234	159–343
11	Morocco (MAR)	55,697	217	147–321
12	Former Yugoslav Republic of Macedonia (MKD)	48,829	193	129–288
13	Lebanon (LBN)	47,718	189	126–283
14	Nigeria (NGA)	34,443	140	91–216
15	Peru (PER)^a^	32,645	133	86–207
16	Indonesia (IDN)	29,920	123	79–193
17	Democratic Republic of the Congo (COD)	22,579	95	59–153
18	Uganda (UGA)	18,139	78	47–128
19	Iraq (IRQ)	16,202	70	42–117
20	Zambia (ZMB)	15,607	68	41–113
21	Mozambique (MOZ)	14,566	64	38–107
22	Angola (AGO)	14,378	63	37–106
23	Jamaica (JAM)	10,698	48	28–83
24	Myanmar (MMR)	10,632	48	28–83
25	Tunisia (TUN)	9,724	44	25–77
26	Bolivia (BOL)	8,959	41	23–72
27	El Salvador (SLV)^a^	6,545	31	17–56
28	Kenya (KEN)	3,153	16	8–31
29	Botswana (BWA)	1,163	6	3–14
30	Senegal (SEN)	799	5	2–10
31	Libya (LBY)	540	3	1–7
32	Bosnia and Herzegovina (BIH)	387	2	1–6
33	Madagascar (MDG)^b^	16	NA	NA
	Total	14,185,272	38,900	36,700–41,100

NA, not applicable.

a Treated as lacking data on mesothelioma frequency because only 2 years of data were available in the WHO mortality database and no other information could be identified (see “Materials and Methods”).

b Out of range of the regression model.
